# Different CFTR modulator combinations downregulate inflammation differently in cystic fibrosis

**DOI:** 10.7554/eLife.54556

**Published:** 2020-03-02

**Authors:** Heledd H Jarosz-Griffiths, Thomas Scambler, Chi H Wong, Samuel Lara-Reyna, Jonathan Holbrook, Fabio Martinon, Sinisa Savic, Paul Whitaker, Christine Etherington, Giulia Spoletini, Ian Clifton, Anil Mehta, Michael F McDermott, Daniel Peckham

**Affiliations:** 1 Leeds Institute of Medical Research at St James's, University of LeedsLeedsUnited Kingdom; 2 Leeds Cystic Fibrosis Trust Strategic Research Centre, University of LeedsLeedsUnited Kingdom; 3 Leeds Institute of Rheumatic and Musculoskeletal Medicine, University of LeedsLeedsUnited Kingdom; 4 Department of Biochemistry, University of LausanneEpalingesSwitzerland; 5 Department of Clinical Immunology and Allergy, St James’s University HospitalLeedsUnited Kingdom; 6 Adult Cystic Fibrosis Unit, St James’s University HospitalLeedsUnited Kingdom; 7 Division of Medical Sciences, University of DundeeDundeeUnited Kingdom; Indian Institute of Science Education and Research (IISER)India; Radboud University Medical CentreNetherlands

**Keywords:** ivacaftor, lumacaftor, tezacaftor, cystic fibrosis, inflammation, nlrp3 inflammasome, Human

## Abstract

Previously, we showed that serum and monocytes from patients with CF exhibit an enhanced NLRP3-inflammasome signature with increased IL-18, IL-1β, caspase-1 activity and ASC speck release (Scambler et al. eLife 2019). Here we show that CFTR modulators down regulate this exaggerated proinflammatory response following LPS/ATP stimulation. In vitro application of ivacaftor/lumacaftor or ivacaftor/tezacaftor to CF monocytes showed a significant reduction in IL-18, whereas IL-1β was only reduced with ivacaftor/tezacaftor. Thirteen adults starting ivacaftor/lumacaftor and eight starting ivacaftor/tezacaftor were assessed over three months. Serum IL-18 and TNF decreased significantly with treatments, but IL-1β only declined following ivacaftor/tezacaftor. In (LPS/ATP-stimulated) PBMCs, IL-18/TNF/caspase-1 were all significantly decreased and IL-10 was increased with both combinations. Ivacaftor/tezacaftor alone showed a significant reduction in IL-1β and pro-IL-1β mRNA. This study demonstrates that these CFTR modulator combinations have potent anti-inflammatory properties, in addition to their ability to stimulate CFTR function, which could contribute to improved clinical outcomes.

## Introduction

Cystic fibrosis (CF) is characterised by repeated pulmonary infections and disordered innate immune-driven inflammation. The relationship between these two key drivers of disease progression remain poorly understood. We and others have recently provided evidence supporting the hypothesis that the NLRP3-inflammasome is a key regulator of inflammation in CF (McElvaney et al., 2019; Peckham et al., 2017; Scambler et al., 2019). We’ve demonstrated that both CF epithelia and monocytes/serum from patients with CF exhibit an enhanced pro-inflammatory signature when compared with healthy controls and patients with non-CF bronchiectasis. We observed CF-specific increases in IL-18, IL-1β, caspase-1 activity, in addition to ASC-speck release, that were all reversed by pre-treatment with epithelial sodium channel (ENaC) and NLRP3-inflammasome inhibitors (Peckham et al., 2017; Scambler et al., 2019). In this paper, we examine the therapeutic potential of controlling inflammation with CFTR modulators. These small molecules are known to correct CFTR dysfunction and partially restore CFTR-mediated ENaC inhibitory activity (Cholon et al., 2014; Pranke et al., 2017).

The NLRP3-inflammasome senses multiple damage-associated molecular patterns (DAMPs) and pathogen-associated molecular patterns (PAMPs). Amongst the latter, lipopolysaccharide (LPS) is a major component of the outer membrane of two of the main pathogens found in CF airways, the gram-negative bacteriae such a *Pseudomonas aeruginosa (P. aeruginosa)* and members of the *Burkholderia cepacia complex (B. cenocepacia)*. It has been reported that the latter organism can trigger the NLRP3-inflammasome and exacerbate the proinflammatory response, both through inflammasome activation and IL-1β/IL-18 processing (Rimessi et al., 2015; Gavrilin et al., 2012; Rosales-Reyes et al., 2012). In this regard, in both murine and human CF, NLRP3 activity contributes to IL-1β-dependent inflammation which can be negatively regulated by anakinra, an IL-1 receptor antagonist (IL-1Ra) (McElvaney et al., 2019; Iannitti et al., 2016). In mice, the NLRP3 inhibitor, MCC950, reduces IL-1β levels in the lungs, decreases airway inflammation and improves airway clearance of *P. aeruginosa* (McElvaney et al., 2019). Thus, defective CFTR expression and function appears to be driving inflammation, by lowering the threshold of innate immune defences and reducing the ability of myeloid cells, such as neutrophils and macrophages to resolve infection and inflammation (Barnaby et al., 2018; Bonfield et al., 2012). Conditional inactivation of *Cftr* in myeloid cells of mice resulted in a dysfunctional immune response, with *Cftr*-lacking mice taking longer to resolve inflammation and to rectify infection, when compared to wild-type (WT) mice (Bonfield et al., 2012). Finally, in young children with CF, inflammation can be detected in bronchoalveolar lavage (BAL) fluid, in the absence of infection, and this process is mainly driven via NLRP3-inflammasome activation (Rosenow et al., 2019; McElvaney et al., 2018).

The introduction of CFTR modulators into clinical practice has resulted in a paradigm shift in therapeutic options, whereby targeting the clinico-pathogenic origin of the defective CFTR rather than the secondary effects of CFTR dysfunction has become a reality (VX17-445-103 Trial Group et al., 2019; VX17-445-102 Study Group et al., 2019; VX08-770-102 Study Group et al., 2011; Taylor-Cousar et al., 2017; TRAFFIC Study Group et al., 2015). These drug regimens were primarily developed to improve cell-surface expression and function of the CFTR anion channel but there are limited data on whether they can directly modulate CF-related inflammation (Barnaby et al., 2018; Hisert et al., 2017). Treatment of primary bronchial epithelial cell cultures (BECs), bearing two Phe508del mutations, with the combination of ivacaftor/lumacaftor (IVA/LUM) depresses CXCL8 transcription, p38 MAPK phosphorylation and reduces serum levels of several proinflammatory cytokines, in response to *P. aeruginosa* (Barnaby et al., 2018; Ruffin et al., 2018). Treatment with the single agent, ivacaftor, currently approved for patients with gating mutations, such as G551D, has also been reported to reduce sputum levels of neutrophil elastase, IL-8, and IL-1β (VX08-770-102 Study Group et al., 2011; Hisert et al., 2017), and these changes may reflect improved clinical health rather than a direct anti-inflammatory consequence of increased CFTR function.

The aims of our study were firstly, to assess whether CFTR modulators could directly downregulate the increased serum and (innate-immune) cell-derived IL-1β and IL-18 cytokine signature in cells harvested from adults with CF, and secondly, to explore differences in response between drug regimens as a guide to inform future studies.

## Results

### Monocyte cytokine responses in healthy controls versus drug-naïve individuals homozygous for Phe508del

**Figure 1.**
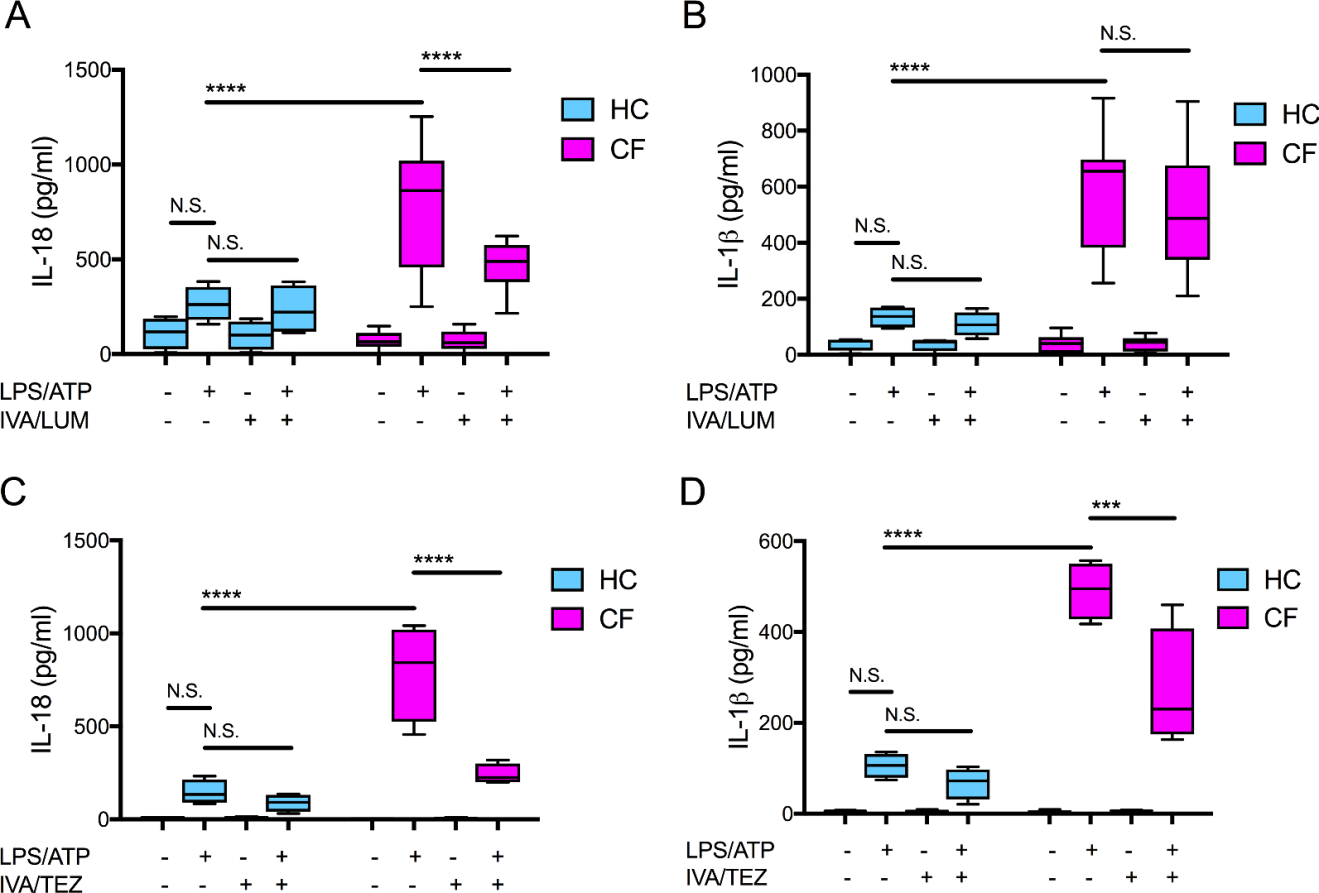
Cytokine secretion in NLRP3-stimulated monocytes with a differential response between CF (homozygous Phe508del) and HC (healthy controls) following in vitro exposure to either IVA/LUM or IVA/TEZ. ELISA assays were used to detect A, C IL-18; B, D IL-1β secretion in monocytes from patients with CF-associated mutations (n = 10 IVA/LUM; n = 4 IVA/TEZ) vs HC (n = 4). Monocytes were treated with IVA/LUM (**A, B**) for 24 hr or IVA/TEZ (**C, D**) for 24 hr, then stimulated with LPS (10 ng/mL, 4 hr), and ATP (5 mM) for the final 30 min. A two-way ANOVA statistical test with Tukey’s multiple comparison was performed (p values * = ≤ 0.05, ** = ≤ 0.01, *** = ≤ 0.001 and **** = ≤ 0.0001). N.S. not-significant. IL-18 levels for HC (no treatment 10.84 pg/ml; IVA/TEZ 11.71 pg/ml); IL-1β levels for HC (no treatment 5.91 pg/ml; IVA/TEZ 5.27 pg/ml). Figure 1—source data 1.Cytokine secretion in NLRP3-stimulated (LPS/ATP) monocytes isolated from CF (homozygous Phe508del) and HC (healthy controls) following in vitro exposure to either IVA/LUM or IVA/TEZ.ELISA assays were used to detect IL-18 or IL-1β secretion (n = 10 IVA/LUM; n = 4 IVA/TEZ) vs HC (n = 4).

We have previously established that monocytes isolated from clinically stable ‘drug-naïve’ CF patients (homozygous for Phe508del) have an increased secretion of IL-18 and IL-1β when compared to healthy control (HC) monocytes. This response was attenuated in vitro with the addition of inhibitors either targeting components of the NLRP3-inflammasome or the ENaC (Scambler et al., 2019). Using monocytes isolated from clinically stable patients homozygous for the common Phe508del CF mutation, we examined whether the in vitro application of clinically approved CFTR modulator combinations (IVA/LUM and IVA/TEZ), could also regulate IL-18 and IL-1β levels. In parallel we established whether these combinations could influence HC cells devoid of pathogenic CFTR mutations. We monitored monocyte stimulated cytokine responses, in cells harvested from drug-naïve patients with CF, in the presence or absence of IVA/LUM or IVA/TEZ combinations. In vitro pre-administration of IVA/LUM for 24 hr to CF monocytes in culture halved the seven-fold rise in IL-18 observed in their absence (p<0.0001, for responses to stimulation with LPS-ATP, Figure 1A). In complete contrast, there was no drug-induced attenuation of the near six-fold rise in LPS-ATP-stimulated IL-1β levels (p=0.9434, Figure 1B). Although HC cells showed a two-fold elevation in cytokines after exposure to LPS-ATP alone, IVA/LUM could not attenuate this small rise (Figure 1A,B). Also, in marked contrast, the in vitro pre-administration of the other drug combination, IVA/TEZ, for 24 hr, significantly reduced the post-stimulus increments in both IL-18 (p<0.0001, Figure 1C) and IL-1β levels (p=0.0001, Figure 1D), in monocytes of patients with CF. IVA/TEZ also had no discernable reductive effect on monocytes from HC volunteers in the presence/absence of LPS and ATP (Figure 1C,D).

### Serum cytokine responses in patients receiving IVA/LUM or IVA/TEZ therapy

Next, we investigated the effects of IVA/LUM or IVA/TEZ CFTR modulator therapy on cytokine secretion in serum from patients homozygous for Phe508del, receiving one or other therapy over a three-month period. Our rationale was that blood is routinely drawn from such patients every few months, thus making any findings much simpler to incorporate into future studies. Thus, blood samples were taken from patients starting IVA/LUM therapy (n = 13) or IVA/TEZ (n = 8) at baseline (pre-therapy), and again at one month and three months of treatment (Supplementary file 1). All patients receiving these therapies met the criteria for the UK compassionate use program, that is reflective of their percent predicted forced expiratory volume (ppFEV_1_) of less than 40 (HC values are typically greater than 80). To ensure maximal clinical stability, patients received a pre-emptive course of intravenous antibiotics prior to starting treatment. Over this three month study period there were no significant differences in C-reactive protein (CRP), ppFEV_1_, forced vital capacity (ppFVC), white blood count (WBC), neutrophil count, weight (kg) or body mass index (BMI) in either group (Supplementary file 1; Figure 2—figure supplement 1 for IVA/LUM; Figure 2—figure supplement 2 for IVA/TEZ).

**Figure 2.**
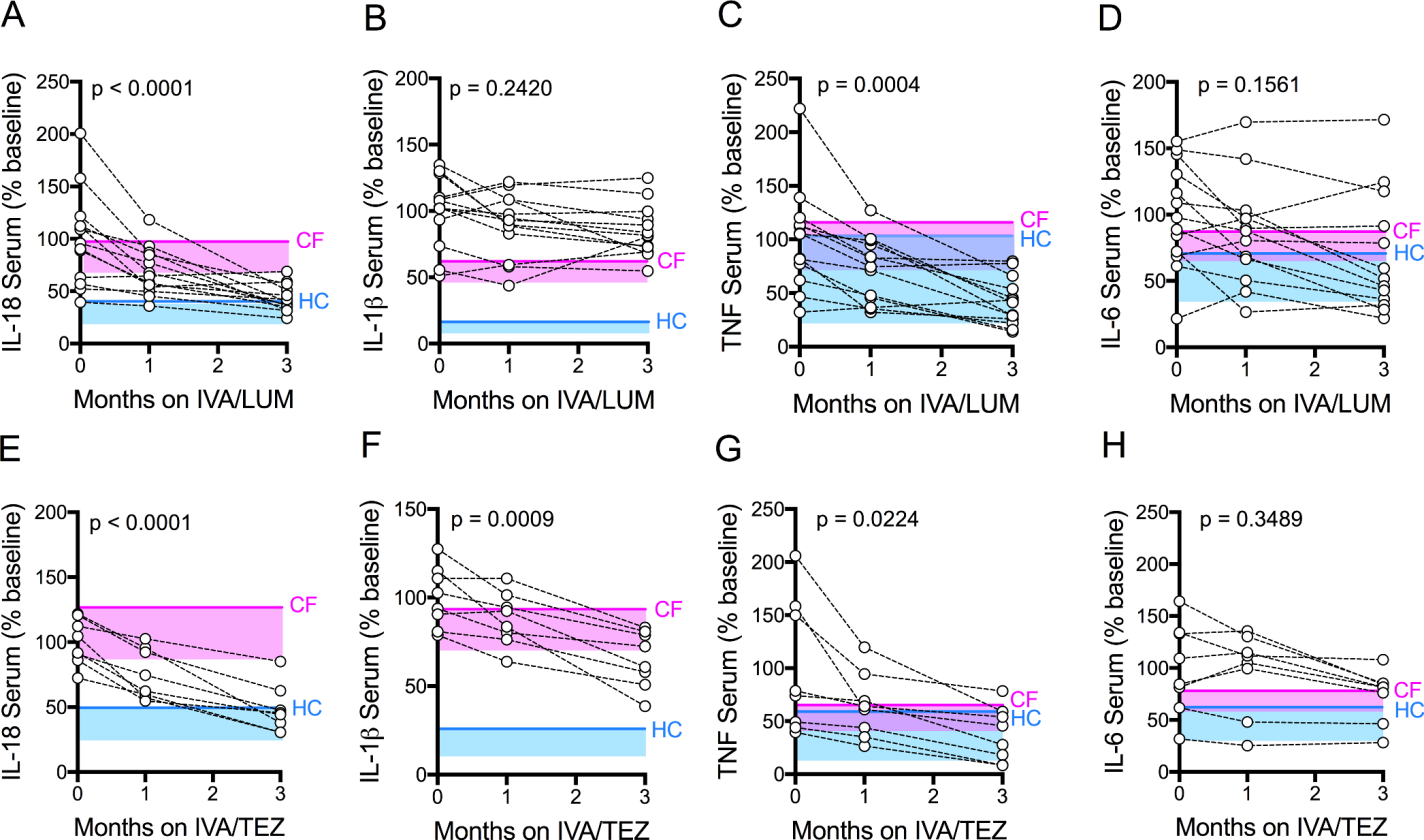
Serum cytokine levels in patients with CF (homozygous Phe508del), following treatment with IVA/LUM or IVA/TEZ. Sera were collected at baseline, one month and three months of treatment from patients homozygous for Phe508del CFTR mutations receiving compassionate use IVA/LUM (n = 13) or IVA/TEZ (n = 8) therapy. ELISA assays were used to detect levels of **A**, IL-18; **B**, IL-1β; **C**, TNF and **D**, IL-6 in serum from the IVA/LUM treated group. ELISA assays were used to detect levels of **E**, IL-18; **F**, IL-1β; **G**,TNF; **H**, IL-6; in serum from the IVA/TEZ treated group. A one-way ANOVA statistical test with Tukey’s multiple comparison was performed. P value for baseline to three months shown on each graph. Baseline ranges were established for each cytokine from HC and clinically stable drug-naïve CF patients (Figure 2—figure supplement 3). Upper 95% confidence interval for baseline HC (solid blue line) or CF (solid pink line) with block colour shading (HC, blue; CF, pink) to lower 5% confidence interval is displayed for each cytokine. Figure 2—source data 1.Serum cytokine levels in patients with CF (homozygous Phe508del), following treatment with IVA/LUM or IVA/TEZ.The baseline (pre-therapy, zero month) values for each patient were calculated as a percentage of the average baseline within each patient group (IVA/LUM or IVA/TEZ). The one month and three month samples were calculated as a percentage of the baseline average. ELISA assays were used to detect IL-18, IL-1β, TNF or IL-6 secretion (n = 13 IVA/LUM; n = 8 IVA/TEZ).

**Figure 3.**
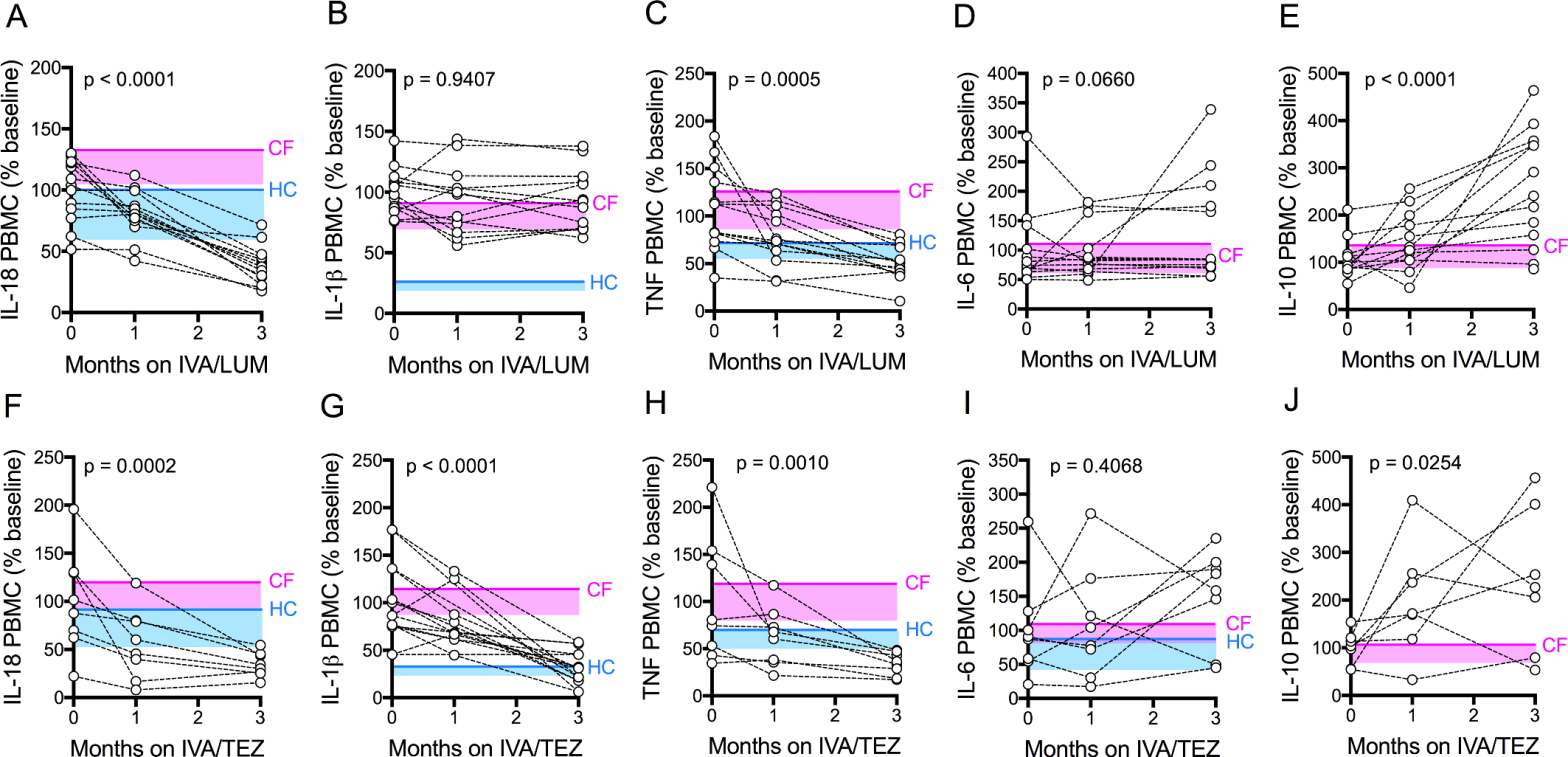
Cytokine secretion in NLRP3-stimulated CF immune cells isolated from patients with CF (homozygous Phe508del), following treatment with IVA/LUM or IVA/TEZ. PBMCs isolated at baseline, one month and three months from patients homozygous for Phe508del CFTR mutations receiving compassionate use IVA/LUM (n = 12/13) or IVA/TEZ (n = 8) therapy. Following isolation, PBMCs were immediately stimulated with LPS (10 ng/mL, 4 hr), and ATP (5 mM) for the final 30 min. ELISA assays were used to detect levels of **A**, IL-18; **B**, IL-1β; **C**, TNF; **D**, IL-6 and **E**, IL-10 secretion from PBMCs in the IVA/LUM treated group. ELISA assays were used to detect levels of **F**, IL-18; **G**, IL-1β; **H**,TNF; **I**, IL-6; **J**, IL-10 secretion from PBMCs in the IVA/TEZ treated group. For IVA/LUM: IL-18 (n = 13); IL-1β (n = 13); TNF (n = 12); IL-6 (n = 13); IL-10 (n = 12). A two-way ANOVA statistical test with Tukey’s multiple comparison was performed. P value for baseline to three months is shown on each graph. For IL-6 (IVA/LUM), a non-parametric Kruskal-Wallis statistical test with Dunn’s multiple comparison was performed. Baseline ranges were established for each cytokine from HC and clinically stable drug-naïve CF patients (Figure 2—figure supplement 3). Upper 95% confidence interval for baseline HC (solid blue line) or CF (solid pink line) with block colour shading (HC, blue; CF, pink) to lower 5% confidence interval is displayed for each cytokine. Figure 3—source data 1.Cytokine secretion in NLRP3-stimulated (LPS/ATP) CF immune cells isolated from patients with CF (homozygous Phe508del), following treatment with IVA/LUM or IVA/TEZ.The baseline (pre-therapy, zero month) values for each patient were calculated as a percentage of the average baseline within each patient group (IVA/LUM or IVA/TEZ). The one month and three month samples were calculated as a percentage of the baseline average. ELISA assays were used to detect IL-18, IL-1β, TNF, IL-6 or IL-10 secretion (n = 13 IVA/LUM; n = 8 IVA/TEZ).

To obtain normal ranges for serum cytokines, baseline ranges were established from 10 HC and 51 clinically stable drug-naïve CF patients (with ppFEV_1_ ranging from 12 to 90; Figure 2—figure supplement 3A–D). From these data, the 95% confidence interval generated upper limits of healthy control values and equivalent CF values which were plotted as horizontal solid blue and pink lines, respectively, with shaded areas extending to the lower 5% confidence interval in Figures 2 and 3, (A-D). The baseline (pre-therapy, zero month) values for each patient were calculated as a percentage of the average baseline within each patient group (IVA/LUM or IVA/TEZ), which explains why the starting values do not all congregate at 100% in the graphs. The one month and three month samples are calculated as a percentage of the baseline average.

**Figure 2—figure supplement 1.**
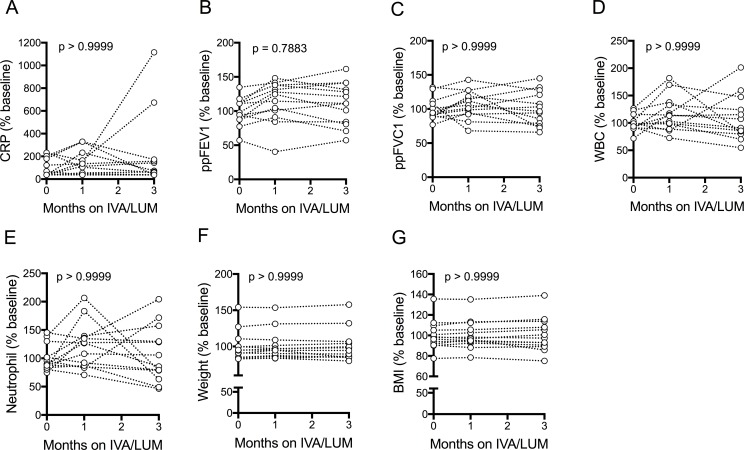
Clinical measurements of patients with CF (homozygous Phe508del) following IVA/LUM treatment. Clinical measurements were taken at baseline, one month and three months from patients homozygous for Phe508del CFTR mutations receiving compassionate use IVA/LUM (n = 13). Measurements were plotted as % baseline. **A**, CRP; **B**, ppFEV1; **C**, ppFVC1; **D**, WBC; **E**, Neutrophil; **F**, Weight; **G**, BMI. A non-parametric Kruskal-Wallis statistical test with Dunn’s multiple comparison was performed. P value for baseline to three months is shown on each graph.

**Figure 2—figure supplement 2.**
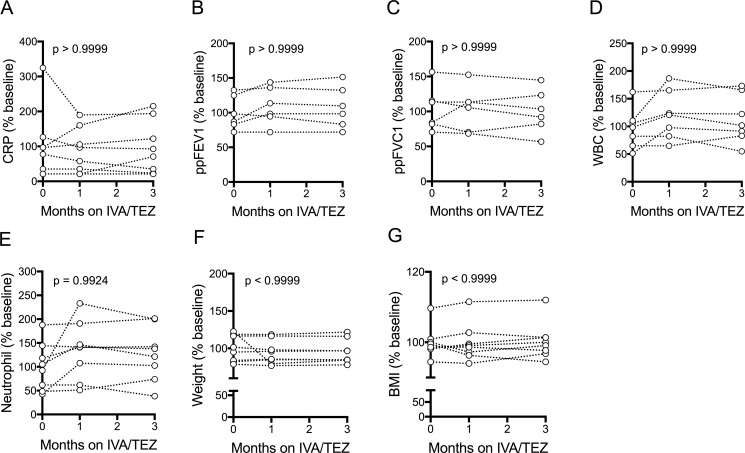
Clinical measurements of patients with CF (homozygous Phe508del) following IVA/TEZ treatment. Clinical measurements were taken at baseline, one month and three months from patients homozygous for Phe508del CFTR mutations receiving compassionate use IVA/TEZ therapy. Measurements were plotted as % baseline. **A**, CRP (n = 8); **B**, ppFEV1 (n = 6); **C**, ppFVC1 (n = 6); **D**, WBC (n = 7); **E**, Neutrophil (n = 8); **F**, Weight (n = 8); **G**, BMI (n = 8). A non-parametric Kruskal-Wallis statistical test with Dunn’s multiple comparison was performed. P value for baseline to three months is shown on each graph.

**Figure 2—figure supplement 3.**
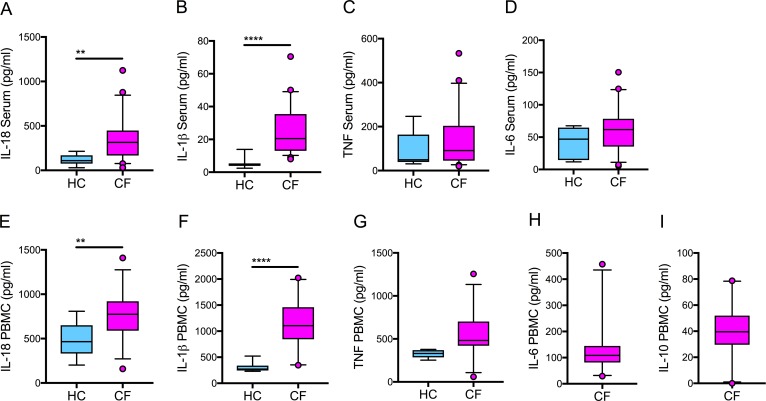
Baseline serum cytokine levels in HC and CF (homozygous Phe508del) and corresponding baseline cytokine levels in NLRP3-stimulated HC and CF immune cells. Serum collected and PBMCs isolated at baseline from patients homozygous for Phe508del CFTR mutations (HC, n = 10; CF, n = 51). ELISA assays were used to detect levels of **A**, IL-18; **B**, IL-1β; **C**, TNF and **D**, IL-6 in serum. ELISA assays were used to detect levels of **E**, IL-18; **F**, IL-1β; **G**,TNF; **H**, IL-6; **I**, IL-10 secretion in PBMCs. PBMCs were stimulated with LPS (10 ng/mL, 4 hr), and ATP (5 mM) for the final 30 min. For PBMC data IL-18, IL-1β (HC n = 11, CF n = 32); TNF (HC n = 6, CF n = 27); IL-6 and IL-10 (CF n = 20) A non-parametric Mann Whitney test was performed. Error bars displayed as 5–95% percentile range with median (p values * = ≤ 0.05, ** = ≤ 0.01, *** = ≤ 0.001 and **** = ≤ 0.0001).

**Figure 2—figure supplement 4.**
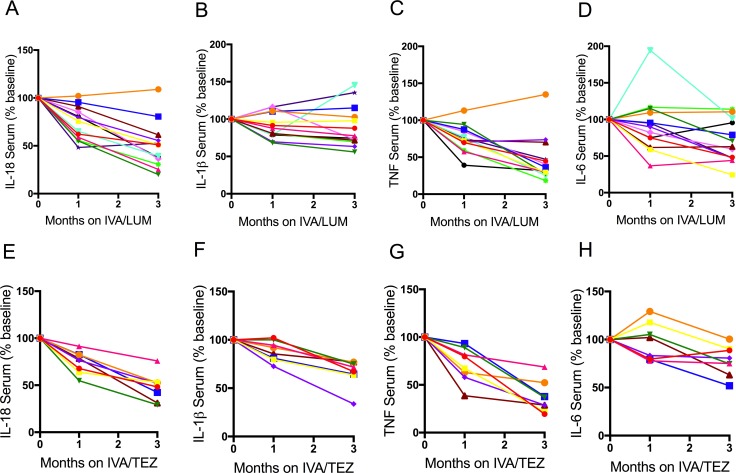
Individual therapy serum cytokine response in patients with CF (homozygous Phe508del) following IVA/LUM or IVA/TEZ treatment. Serum collected at baseline, one month and three months from patients homozygous for Phe508del CFTR mutations receiving compassionate use IVA/LUM (n = 13) or IVA/TEZ (n = 8) therapy. ELISA assays were used to detect **A**, IL-18; **B**, IL-1β; **C**, TNF and **D**, IL-6 in serum from the IVA/LUM treated group. ELISA assays were used to detect levels of **E**, IL-18; **F**, IL-1β; **G**,TNF; **H**, IL-6; in serum from the IVA/TEZ treated group. Baseline for each patient set at 100% and proportional change at one and three months plotted.

**Figure 3—figure supplement 1.**
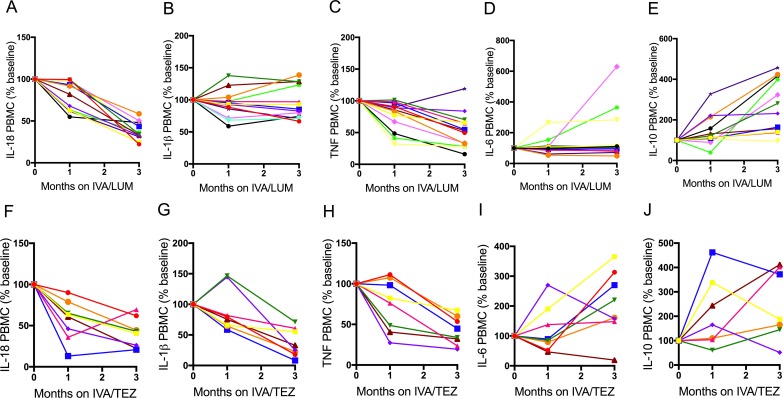
Individual therapy PBMC cytokine response in NLRP3- stimulated CF immune cells isolated from patients with CF (homozygous Phe508del) following treatment with IVA/LUM or IVA/TEZ. PBMCs isolated at baseline, one month and three months from patients homozygous for Phe508del CFTR mutations receiving compassionate use IVA/LUM (n = 12/13) or IVA/TEZ (n = 8) therapy. Following isolation, PBMCs were immediately stimulated with LPS (10 ng/mL, 4 hr), and ATP (5 mM) for the final 30 min. ELISA assays were used to detect levels of **A**, IL-18; **B**, IL-1β; **C**, TNF; **D**, IL-6 and **E**, IL-10 secretion from PBMCs in the IVA/LUM treated group. ELISA assays were used to detect levels of **F**, IL-18; **G**, IL-1β; **H**,TNF; **I**, IL-6; **J**, IL-10 secretion from PBMCs in the IVA/TEZ treated group. Baseline for each patient set at 100% and proportional change at one and three months plotted. For IVA/LUM: IL-18 (n = 13); IL-1β (n = 13); TNF (n = 12); IL-6 (n = 13); IL-10 (n = 12).

There was a significant reduction in serum IL-18 levels, following three month’s treatment with either IVA/LUM (p<0.0001, Figure 2A) or IVA/TEZ (p<0.0001, Figure 2E). The net effect was a final IL-18 cytokine level lower than the CF upper limit for both groups. Interestingly, values bordering on the upper limit of HC values were mainly seen after IVA/TEZ. However, marked drug-dependent differences between cytokine responses were also observed. There was no decline in serum IL-1β levels after three months therapy with IVA/LUM (Figure 2B), and yet IVA/TEZ treatment resulted in a highly significant reduction in serum IL-1β (p=0.0009, Figure 2F; compare 2B). This differential response was mirrored with the NLRP3-inflammasome-independent cytokines which, in both treatment groups, showed a significant reduction in serum TNF (IVA/LUM, p=0.0004, Figure 2C; IVA/TEZ, p=0.0224, Figure 2G) but no change in serum IL-6 levels (Figure 2D and H).

To establish the individual response to therapy (IVA/LUM or IVA/TEZ), serum cytokine baselines were set to 100% for each patient, and the percentage change at one month and three months post-therapy were displayed (Figure 2—figure supplement 4). We observed that there were fast (<25% decrease from baseline) and slow responders (<10% decrease from baseline) at one-month post-therapy for each cytokine; however, the individual therapy response for each cytokine was different, and no correlation between individuals was observed at this time point. Although IVA/LUM therapy significantly decreased IL-18 and TNF levels in serum (Figure 2A,C), the percentage to baseline response at three months revealed a broad range of effectiveness (IL-18; 109–19%; TNF 135–18%) (Figure 2—figure supplement 4A,C), whereas for IVA/TEZ therapy, at three months, the minimum percentage decrease from baseline was 25% for IL-18, IL-1β and TNF cytokine measurements (IL-18 75–29%; IL-1β 74–33% and TNF 68–19%) (Figure 2—figure supplement 4, E–G) suggesting that overall, the therapy response to IVA/TEZ is more consistent across this patient group.

### PBMC cytokine response following LPS and ATP stimulation in patients receiving IVA/LUM or IVA/TEZ therapy

Next, we investigated the above differences at a cellular level, initially using unstimulated peripheral blood monocytes (PBMC) isolated from CF patients. Our rationale was that the isolation procedures for such cells might be easier to incorporate into future trials when compared to the more cumbersome monocyte isolation protocol. Generally, levels ranged below 50 pg/ml, irrespective of exposure to either of the two drug combinations. There were no significant changes in the levels of pro-inflammatory cytokines IL-18, IL-1β, TNF, IL-6 and IL-10 secretion by unstimulated PBMCs, at baseline, or at one or three months of treatment in patients receiving either IVA/LUM or IVA/TEZ (Supplementary file 2).

Next, we stimulated the baseline CF-PBMC’s with LPS and ATP. Prior to starting IVA/LUM or IVA/TEZ, at baseline (pre-therapy), the LPS-ATP stimulated PBMCs secreted between ten-and fifty- fold greater amounts of IL-18 (IVA/LUM, 642 pg/mL; IVA/TEZ, 719 pg/mL), IL-1β (IVA/LUM 1422 pg/mL; IVA/TEZ 1119 pg/mL), TNF (IVA/LUM, 517 pg/mL; IVA/TEZ, 568 pg/mL), IL-6 (IVA/LUM, 156 pg/mL; IVA/TEZ, IL-6 (217 pg/mL) and IL-10 (IVA/LUM, 37 pg/ml; IVA/TEZ 47 pg/ml). These baseline values are consistent with the mean values from a larger, clinically stable drug-naïve patient group (Figure 2—figure supplement 3, E–I). HC measurements, for IL-6 and IL-10 in the LPS-ATP stimulated PBMCs were not taken for the larger drug-naïve patient group and, therefore, data are not shown.

After three months of patient treatment with oral therapy combinations, LPS-ATP stimulated PBMC secretion of IL-18 was significantly reduced (by approximately three-fold), compared to baseline, for both IVA/LUM (247 pg/mL p =< 0.0001, Figure 3A) and IVA/TEZ (247 pg/mL p=0.0002, Figure 3F). Interestingly, and similar to the serum cytokine profiles, after PBMC stimulation, IL-1β remained unchanged in cells exposed in vivo to IVA/LUM (Figure 3B) after PBMC stimulation, but fell significantly in those patients receiving IVA/TEZ (378 pg/mL, p<0.0001, Figure 3G).

Following oral treatment, stimulated PBMC TNF secretion was also significantly reduced at three months, compared to baseline, for both IVA/LUM (217 pg/mL p=0.0005, Figure 3C) and IVA/TEZ (193 pg/mL, p=0.001, Figure 3H) but IL-6 levels remained unchanged for both drug combinations (Figure 3D and I). Interestingly, levels of the anti-inflammatory cytokine IL-10 significantly increased at three months, post-stimulation, compared to baseline, for both IVA/LUM (95 pg/mL, p<0.0001, Figure 3E); and IVA/TEZ (112 pg/mL, p=0.025, Figure 3J).

When we examined the individual patient response to therapy (IVA/LUM or IVA/TEZ) in LPS-ATP-stimulated PBMCs, we observed a range of cytokine responses at one month post-therapy in a similar manner to the serum response, and the individual therapy responses for each cytokine was different, and again no correlation between individuals was observed at this time point (Figure 3—figure supplement 1). However, in contrast to the serum cytokine profiles which showed a broad range of effectiveness for IL-18, LPS-ATP-stimulation of PBMCs following IVA/LUM therapy at three months, was found to produce a minimum percentage decrease from baseline of 31% for IL-18 but TNF remained similar to the response observed in serum (IL-18 59–22%; TNF 119–16%) (Figure 3—figure supplement 1A,C). Interestingly, LPS-ATP-stimulated PBMCs, post IVA/TEZ therapy, mirrored the cytokine profiles observed at three-months in serum, with the minimum percentage decrease from baseline of 29% for IL-18, IL-1β and TNF cytokines measurements (IL-18 69–20%; IL-1β 71–8% and TNF 67–19%) (Figure 3—figure supplement 1F–H).

### NLRP3-inflammasome activation in patients receiving IVA/LUM or IVA/TEZ therapy

**Figure 4.**
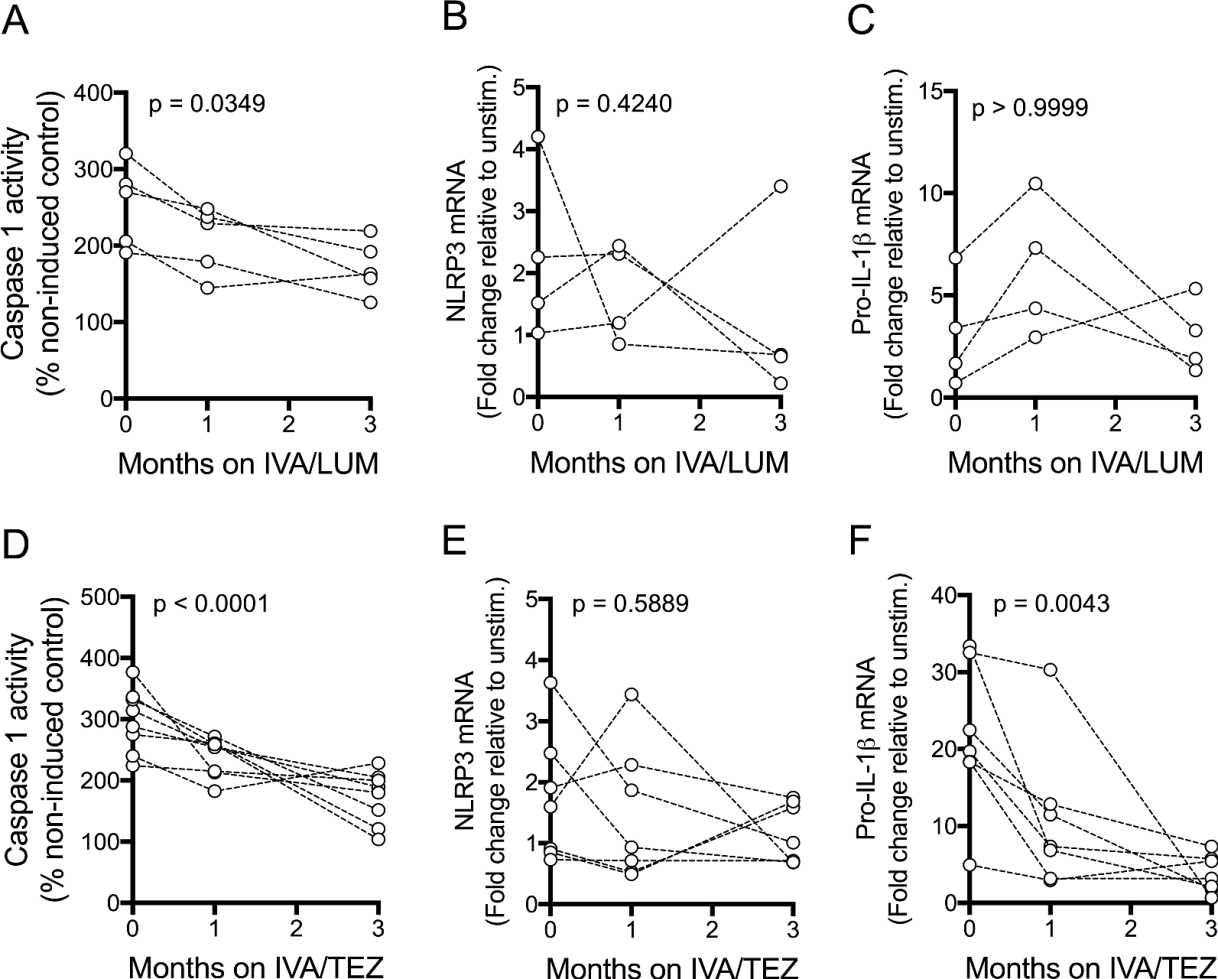
NLRP3-inflammasome activation in patients with CF (homozygous Phe508del) receiving IVA/LUM or IVA/TEZ treatment. PBMCs isolated at baseline, one month and three months from patients homozygous for Phe508del CFTR mutations receiving compassionate use IVA/LUM or IVA/TEZ therapy. Following isolation, PBMCs were immediately stimulated with LPS (10 ng/mL, 4 hr), and ATP (5 mM) for the final 30 min. Caspase-1 activity was detected in stimulated PBMCs at each time point for **A**, IVA/LUM (n = 4) and D, IVA/TEZ (n = 8). qPCR analysis was used to asses a fold change in mRNA expression of B, E NLRP3, and C, F pro-IL-1β for IVA/LUM (n = 4) (**B–C**) or IVA/TEZ (n = 7) (**E–F**). A one-way ANOVA with Tukey’s multiple comparison was performed on caspase-1 activity data and a non-parametric Kruskal-Wallis statistical test with Dunn’s multiple comparison was performed on the mRNA data. P value for baseline to three months indicated on each graph.

In a further pilot study, aimed at assessing possible reasons for the differential drug responses seen above, caspase-1 activity and mRNA levels were monitored in a subset of patients receiving IVA/LUM (n = 4) and IVA/TEZ (n = 8/7). Serum caspase-1 activation was reduced following three months of IVA/LUM (p=0.0349, Figure 4A) and IVA/TEZ therapy (p<0.0001, Figure 4D). At three months, a significant decrease in mRNA levels of *pro-IL-1β* was only seen with IVA/TEZ (p<0.0001, Figure 4F) and not with IVA/LUM. There were no significant differences in *NLRP3* (Figure 4B) or *TNF* transcript levels, from baseline to three months, in the IVA/LUM group, and there was a non-significant trend to increased mRNA transcript levels, both *IL-6* and *IL-10* (Figure 4—figure supplement 1, B–D).

**Figure 4—figure supplement 1.**
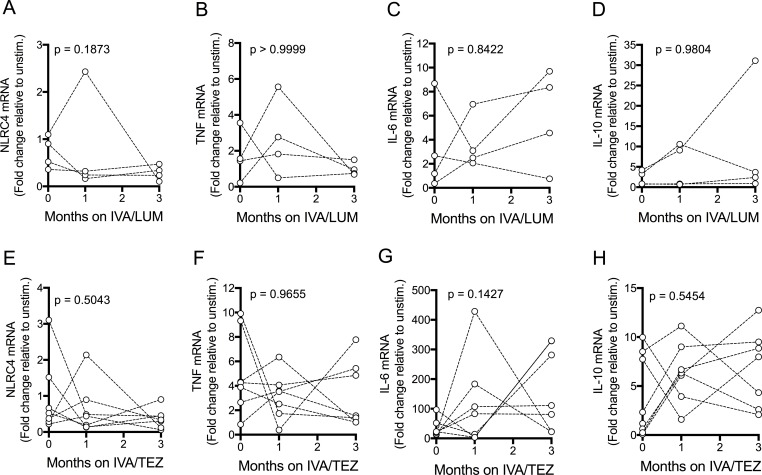
mRNA expression in NLRP3-stimulated PBMCs isolated from patients with CF (homozygous Phe508del) following IVA/LUM or IVA/TEZ treatment. PBMCs isolated at baseline, one month and three months from patients homozygous for Phe508del CFTR mutations receiving compassionate IVA/LUM or IVA/TEZ treatment. Following isolation, PBMCs were immediately stimulated with LPS (10 ng/mL, 4 hr), and ATP (5 mM) for the final 30 min. qPCR analysis was used to asses a fold change in mRNA expression of A, E NLRC4, B, F TNF, C, G IL-6, D, H IL-10 for IVA/LUM (n = 4) (**A–D**) or IVA/TEZ (n = 7) (**E–H**). A non-parametric Kruskal-Wallis statistical test with Dunn’s multiple comparison was performed on mRNA data. P value for baseline to three months indicated on each graph.

In the IVA/TEZ group, mRNA levels of *IL-6* mRNA showed a non-significant trend to increased levels (p=0.0595, Figure 4—figure supplement 1, G), from baseline to three months, and there was a non-significant decrease in *NLRC4* and *TNF*, plus an increase in *IL-10* mRNA transcripts (Figure 4—figure supplement 1, E,F,H).

### Sustainability of oral therapy (IVA/LUM or IVA/TEZ) on NLRP3-stimulated cytokine production in PBMCs isolated from patients on therapy for three months

**Figure 5.**
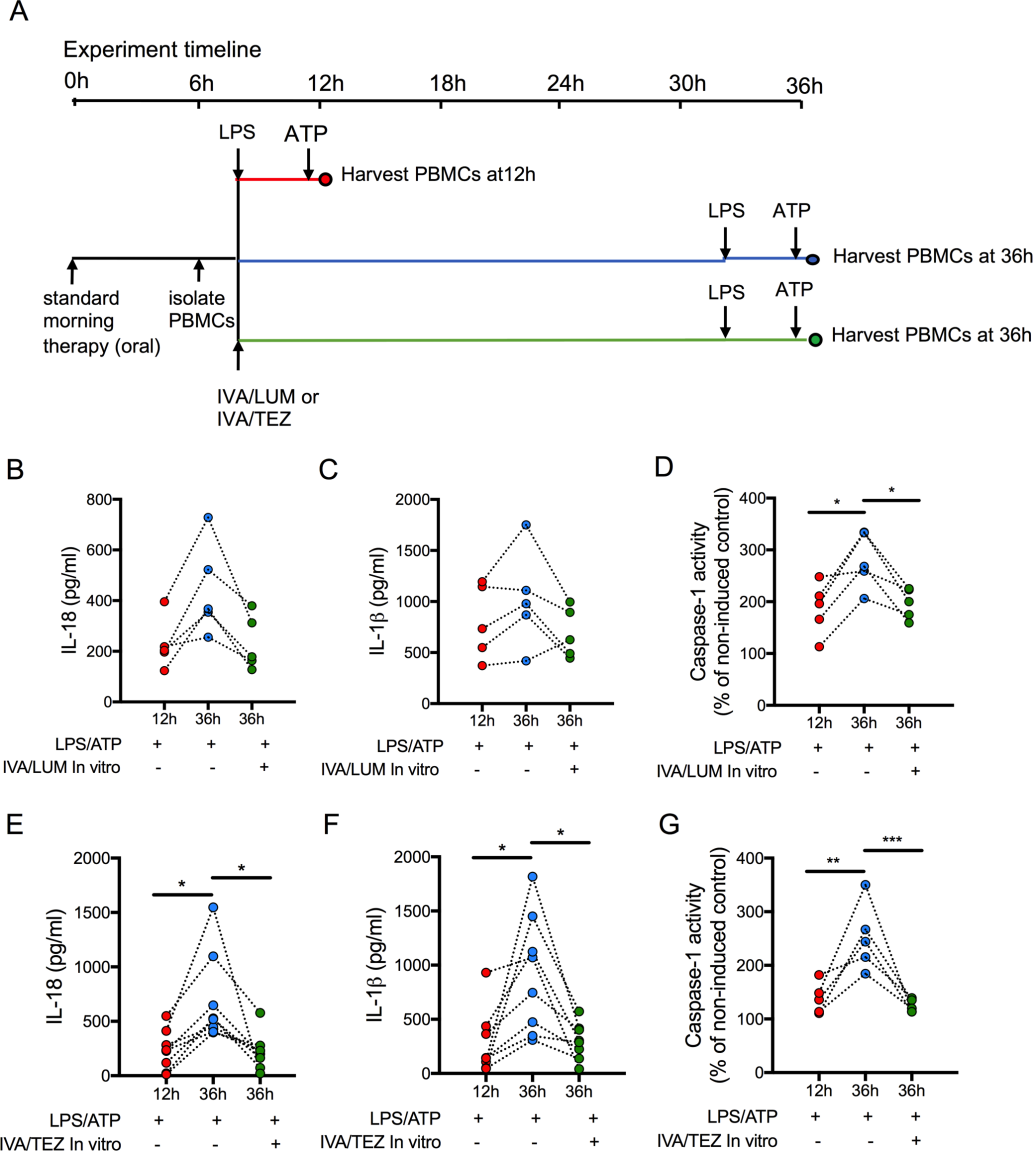
Sustainability of oral drug therapy (IVA/LUM or IVA/TEZ) on NLRP3-stimulated cytokine production in PBMCs isolated from patients on therapy for three months. PBMCs were isolated from patients receiving IVA/LUM or IVA/TEZ treatment for three months or longer. PBMCs were stimulated with LPS (10 ng/mL, 4 hr), and ATP (5 mM) for the final 30 min at 12 hr post oral therapy and at 36 hr post oral therapy with or without pre-treatment with IVA/LUM or IVA/TEZ as shown in schematic **A**. ELISA assays were used to detect levels of (**B, E**), IL-18; (**C, F**), IL-1β; secretion in media from PBMCs (IVA/LUM, n = 5; IVA/TEZ. n = 8). (**D, G**), Caspase-1 activity was detected in stimulated PBMCs at each time point, (IVA/LUM, n = 5; IVA/TEZ, n = 5). A one-way ANOVA with Tukey’s multiple comparison was performed on caspase-1 activity data and a non-parametric Kruskal-Wallis statistical test with Dunn’s multiple comparison was performed on IL-18 and IL-1β data (p values * = ≤ 0.05, ** = ≤ 0.01, *** = ≤ 0.001 and **** = ≤ 0.0001).

Next, we attempted to establish how long oral drug therapy might be sustained in vitro by measuring cytokine levels in stimulated PBMCs at 12 and 36 hr post-oral therapy. We also wished to determine whether re-application of a given drug combination on isolated cells in vitro at 12 hr post-oral therapy would have any effect on stimulated PBMCs. In patients on IVA/LUM (n = 5), caspase-1 activity (p=0.0194, Figure 5D) was significantly reduced at 12 hr post-oral therapy (compared to 36 hr), but the changes in IL-18 (228–446 pg/mL, p=0.0644, Figure 5B) and IL-1β levels (799–1025 pg/mL, p=0.619, Figure 5C) were not significant. Following in vitro exposure of these cells to IVA/LUM there was a decrease in caspase-1 activity at 36 hr (p=0.04) but no significant change in either IL-18 (232 pg/ml, p=0.0707) or IL-1β levels (690 pg/mL, p=0.3661).

In contrast, levels of IL-18 (233–699 pg/mL, p=0.0081, Figure 5E), IL-1β (277–918 pg/mL, p=0.0067, Figure 5F) and caspase-1 (p=0.0021, Figure 5G) were significantly lower at 12 hr post-oral therapy relative to 36 hr, in patients receiving oral IVA/TEZ (n = 8). Following in vitro application of IVA/TEZ, the increased IL-18 (221 pg/mL, p=0.0067), IL-1β (298 pg/mL, p=0.0086) and caspase-1 activity (p=0.0009) was reversed, similar to NLRP3-mediated cytokine secretion of IL-18 and IL-1β, induced by IVA/TEZ following oral therapy (Figure 3F,G).

## Discussion

There is an ongoing debate as to whether correcting the primary ion transport defect, with CFTR modulators alone, can resolve both infection and inflammation, two key components driving CF lung pathology. As a result several novel anti-inflammatory agents are now being developed with the aim of slowing down disease progression (Cystic Fibrosis Foundation, 2020). As recurrent episodes of CF inflammation are the direct result of CFTR dysfunction, it follows that correcting CFTR itself should influence the aberrant changes in innate immunity present in CF, and downregulate the self-propagating inflammatory response (Mesureur et al., 2017).

The present study provides evidence of differential downstream regulation of inflammation, in vivo, following short term administration of IVA/LUM or IVA/TEZ. We observed a direct effect of CFTR modulators in both serum and NLRP3-stimulated PBMCs. Treatment with either IVA/LUM or IVA/TEZ significantly reduced serum and NLRP3-stimulated IL-18levels, TNF secretion and caspase-1 activity. There was also an increase in serum IL-10, which is a potent anti-inflammatory cytokine that downregulates the inflammatory response and ameliorates immunopathology (Asadullah et al., 2003). Only IVA/TEZ significantly reduced serum and NLRP3-stimulated IL-1β secretion and *pro-IL-1β,* mRNA transcripts. This difference is potentially important, as IL-1β is significantly elevated in CF and has a wide range of biological effects, associated with both infection and inflammation. Similar differences were also seen following the addition of IVA/TEZ drug to monocytes isolated from clinically stable ‘drug-naïve’ CF patients, homozygous for Phe508del.

Both LUM and TEZ exert their clinical effects by increasing the processing and trafficking of mature CFTR protein to the cell surface. When combined with the potentiator, IVA, they partially rectify CFTR function and alter CFTR-ENaC coupling, which results in the inhibition of the elevated amiloride-sensitive sodium transport that is characteristic of CF (Cholon et al., 2014; Pranke et al., 2017). Studies of nasal potential differences and rectal intestinal current measurements (ICM) studies suggest that dual combination therapy rescue Phe508del CFTR, by approximately 10% to 18% of normal, with both drugs having a similar effect on ion transport (Gentzsch and Mall, 2018; Graeber et al., 2018). The disparity in IL-1β secretion was, therefore, surprising and may result from off-target effects, which have been reported with CFTR modulators. For instance, IVA can influence various solute carriers in vitro and reduce the stability of LUM-rescued Phe508del-CFTR (Chin et al., 2018). Clinically, IVA/LUM has also been associated with unexplained increased respiratory adverse events, which can occur relatively acutely after starting treatment. Symptoms include dyspnoea and chest tightness, features which have not been reported with IVA/TEZ (Habib et al., 2019). It might also be the case that pharmacokinetics of LUM and TEZ may differ.

During this study, combination therapy was only available through a Vertex compassionate use program, with treatment inclusion criteria including lung transplant assessment and a ppFEV1 <40%, for at least two months. This is in contrast to reported phase three clinical trials which recruited stable patients with significantly higher lung function. In those studies, treatment over a 24 week period resulted in modest but significant improvements in lung function of around 3%, with reduced pulmonary exacerbations and a decrease in annual rate of ppFEV1 decline vs matched controls (Konstan et al., 2016). Furthermore, in adults with severe obstructive lung disease receiving IVA/LUM, significant improvements in FEV_1_ can be absent in the first six months of treatment (Wark et al., 2019). While our study was not designed to investigate clinical efficacy, the absence of significant changes in clinical parameters, during our three month study period, was consistent with stable disease rather than a decline in health, as might be expected in a population with severe lung disease.

The association between CF and increased secretion of both IL-18 and IL-1β, as well as *pro-IL-1β* mRNA transcripts, may suggest that inhibiting IL-1β alone, with drugs such as anakinra, an IL-1 receptor antagonist, may prove less effective than drugs which target CFTR and, thereby, modify both IL-18 and IL-1β secretion. However baseline IL-1β levels remained elevated and additional targeting of the IL-1 receptor, could significantly enhance clinical effect, especially in patients on IVA/LUM where IL-1β was not significantly downregulated.

The effect of IVA/LUM and IVA/TEZ was lost at 36 hr post-oral therapy, but was regained following re-incubation with the corresponding drugs in vitro. This suggests that the anti-inflammatory properties of CFTR modulators can be prolonged, when drug levels are sustained through in vitro administration, but also highlights the additional effect of CFTR modulators on pro-inflammatory cytokine levels. This finding has potential implications for patients who struggle with compliance, as missing a single dose will influence inflammatory cytokine levels, and may lead to unwanted side-effects as well as influencing the effectiveness of the therapy (Abbott and Bilton, 2015).

From this study, it seems that IL-18 and IL-1β are the most reliable biomarkers to inform drug effectiveness at downregulating inflammation in CF. Although TNF is not elevated in serum or PBMCs from CF relative to HC (Figure 2—figure supplement 2, C), it has been shown by independent research groups, that the CFTR is actively involved in the regulation of TNF, via the NF- κB pathway in lung epithelial cells (Hunter et al., 2010; Vij et al., 2009). For example, transient-transfection of WT CFTR into CFTR-naive H441 lung epithelial cells, dose-dependently down-regulates both the basal and TNF evoked NF-κB activity (Hunter et al., 2010).

Some consideration should also be given to other biomarkers, such as IL-6, which did not change significantly with treatment and to the anti-inflammatory IL-10 cytokine which rose significantly and may help discriminate individual and drug specific responses in future studies.

In summary, systemic inflammation plays a major role in the pathogenesis of CF and, to our knowledge, this pilot study is the first to demonstrate that CFTR modulators have potent innate anti-inflammatory properties, that can be measured in the clinic, both ex vivo and in vitro. Differences in drug effects on regulation of the inflammatory response highlight the importance of optimising biomarkers of inflammation, when assessing their individual treatment efficacy, which can substantially vary between patients. The introduction of newer, and more effective, drug combinations may prove highly efficacious at normalising the inflammatory response in CF, with the prospect of controlling disease progression. We believe our approach creates a template for a candidate cytokine protocol for future evaluation in CF trials of dysregulated inflammation in CF.

## Materials and methods

**Key resources table:** 

Reagent type (species) or resource	Designation	Source or reference	Identifiers	Additional information
Biological sample (*Homo sapiens*)	Human Blood Samples	St James's University Hospital	Health Research Authority REC reference 17/YH/0084	
Chemical compound, drug	Lymphoprep	Axis Shield	Cat# 1114544	
Chemical compound, drug	Pan Monocyte Isolation Kit, human	Miltenyi Biotec	Cat# 130-096-537	
Chemical compound, drug	Lipopolysacchride Ultrapure EK	InvivoGen	Cat# tlrl-eklps	10ng/ml
Chemical compound, drug	ATP	InvivoGen	Cat# tlrl-atpl	5 mM, 30 min
Chemical compound, drug	Lumacaftor (LUM)	AdooQ Bioscience	Cat#A10986	3 μM, 24 hr
Chemical compound, drug	Ivacaftor (IVA)	Cayman chemicals	Cat#15145	5 μM, 24 hr
Chemical compound, drug	Tezacaftor (TEZ)	TargetMol	Cat#T2263	5 μM, 24 hr
Commercial assay or kit	IL-1 beta Human Matched Antibody Pair	ThermoFisher Scientific	Cat# CHC1213	Assay sensitivity < 31.2 pg/mL
Commercial assay or kit	IL-18 Human Matched Antibody Pair	ThermoFisher Scientific	Cat# BMS267/2MST	Assay sensitivity 78 pg/mL
Commercial assay or kit	IL-6 Human Matched Antibody Pair	ThermoFisher Scientific	Cat# CHC1263	Assay sensitivity 15.6 pg/mL
Commercial assay or kit	TNF alpha Human Matched Antibody Pair	ThermoFisher Scientific	Cat# CHC1753	Assay sensitivity < 15.6 pg/mL
Commercial assay or kit	IL1RA Human Matched Antibody Pair	ThermoFisher Scientific	Cat# CHC1183	Assay sensitivity < 31.2 pg/mL
Chemical compound, drug	(TMB) substrate solution	Sigma	Cat# T0440	
Commercial assay or kit	Caspase-1 Colorimetrix Assay	R and D Systems	Cat# BF15100	
Commercial assay or kit	High-Capacity cDNA Reverse Transcription Kit	ThermoFisher Scientific	Cat# 4368814	
Software, algorithm	GraphPad Prism7	Graphpad software		

### Patients

Adult patients entering the Vertex compassionate use program for IVA/LUM (n = 13) and IVA/TEZ (n = 8) were prospectively recruited between 2016 and 2019, from the Leeds Regional Adult CF Unit. Those on continued treatment for three months were included in the analyses. All subjects on combination therapy were homozygous for Phe508del, had a ppFEV_1_ <40% and had received a course of intravenous antibiotics prior to starting treatment. Lung function, weight, BMI, CRP, WBC, serum cytokines were all measured at baseline, and again at one and three months of treatment (Supplementary file 1). Venous blood for peripheral blood mononuclear cell (PBMC) assays was taken at the same time points. The study was approved by Yorkshire and The Humber Research Ethics Committee (17/YH/0084).

### Samples

Patients’ bloods were collected, using Vacuette tubes (Greiner-Bio-One) containing serum clot activator gel or EDTA for whole blood. Bloods in serum clot activator tubes were allowed to clot for 60 min, followed by centrifugation at 1000xg for 10 min. Sera were collected into 1 mL tubes for storage at −80°C.

### Cell culture

PBMCs were isolated from whole blood, using Lymphoprep gradient media (Axis-Shield, Dundee, UK) and cultured in complete RPMI medium (RPMI medium containing 10% heat-inactivated fetal bovine serum, 2 mM L-glutamine, 50 U/mL penicillin, 50 μg/mL streptomycin). PBMCs (2 × 10^6^/ mL) were allowed to settle overnight, prior to experimentation for Figures 1 and 2. NLRP3-inflammasome stimulation was achieved using LPS (10 ng/mL, Ultrapure *Escherichia coli* K12, Invivogen) for 4 hr, with the addition of ATP (5 mM, Invivogen, San Diego, California) for the final 30 min of stimulation.

Monocytes were isolated by negative selection from PBMCs on the same day using the Pan Monocyte Isolation Kit II (Miltenyi Biotec GmbH), and plated at 1 × 10^6^ cells/mL. All cells were kept in a humidified incubator at 37°C, 5% CO_2._

### Cytokine quantification using ELISA

Two pro-inflammatory, NLRP3-associated cytokines, IL-1β and IL-18, were measured, as well three other cytokines also associated with innate immune cell activation, namely IL-6, TNF and IL-10. Levels of IL-18, IL-1β, TNF, IL-6 and IL-10 cytokines were detected by ELISAs from patients’ sera and cell cultured media, using commercially available ELISA kits (ThermoFisher Scientific, Loughborough, UK) as per manufacturers ' recommendations.

### Caspase-1 activity assay

A colorimetric assay (Caspase-1 Colorimetrix Assay, R and D Systems, Abingdon, UK) measured caspase-1 activity, via the cleavage of a caspase-specific peptide conjugated to a colour reporter molecule, p-nitroalinine (pNA), performed on cell lysates. Protein concentrations in lysates were determined using the Pierce bicinchoninic acid (BCA) assay (Thermo Fisher Scientific, Loughborough, UK).

### Detection of mRNA by RT-qPCR

Total RNA isolation was performed using Trizol reagent and the Phasemaker Tubes (ThermoFisher Scientific) according to the manufacturers’ protocol. RNA quality and quantity were further determined by 260/280 and 260/230 ratios using a NanoDrop spectrophotometer. RNA was converted to cDNA, using no more than 1 μg of sample with the High-Capacity cDNA Reverse Transcription Kit (ThermoFisher Scientific). Real-time quantitative PCR was done using PowerUp SYBR Green Master Mix reagent in the QuantStudio 7 Flex Real-Time PCR System (ThermoFisher Scientific) to determine the mRNA levels of the reported genes (*NLRP3, NLRC4, pro-IL-1β*, *TNF, IL-6* and *IL-10*). mRNA levels were normalised to the levels of *HPRT* RNA transcripts. All primers were optimised for specific amplification of the target gene.

### Sustainability of oral therapy (IVA/LUM or IVA/TEZ) on NLRP3-stimulated cytokine production in PBMCs

Blood samples were collected from patients homozygous for Phe508del receiving compassionate use IVA/LUM or IVA/TEZ to determine how long oral drug therapy would be sustained in vitro by measuring cytokine levels in stimulated PBMCs at two time-points (12 hr and 36 hr post oral-therapy), and also to establish whether the in vitro application of each drug could sustain the drug therapy effect. Both patient cohorts received treatment for at least three months and the last dose was given within six hours of blood sampling (Figure 4). PBMCs were isolated, and either immediately stimulated with LPS and ATP (12 hr post-oral therapy) or stimulated with LPS and ATP 36 hr post-oral therapy. In parallel, PBMCs were treated at 12 hr post-oral therapy in vitro with 3 μM LUM/5 μM IVA (lumacaftor; cat#A10986, AdooQ Bioscience, Generon, Slough; cat#15145, Cayman chemicals, Michigan), or 5 μM TEZ/5 μM IVA (Tezacaftor; cat#T2263, TargetMol, MA, USA), respectively, and stimulated with LPS and ATP 36 hr post-treatment.

### Statistical analyses

All analyses were performed using GraphPad Prism v 7. Shapiro-Wilk test was performed to determine if data was normally distributed for all data sets. The Kruskal-Wallis test, with Dunn’s multiple comparison or Mann Whitney test was performed when comparing non-parametric populations. A one-way ANOVA or two-way ANOVA statistical test, with Tukey’s multiple comparison was performed when calculating variance between samples with a normal distribution (p values *≤0.05, **≤0.01, ***≤0.001 and ****≤0.0001). A p<0.05 was considered significant).

## Data Availability

All data generated or analysed during this study are included in the manuscript and supporting files.

## Additional files

Supplementary file 1. Demographic and clinical characteristics for CF patients on ivacaftor/lumacaftor (IVA/LUM) and ivacaftor/tezacaftor (IVA/TEZ). Data are expressed as median and range. BMI: Body Mass Index; ppFEV: percent predicted forced expiratory volume, ppFVC: forced vital capacity, CRP: C-reactive protein. WBC: white blood count.

Supplementary file 2. Cytokine secretion in unstimulated CF PBMCs following IVA/LUM (n = 13) or IVA/TEZ (n = 8) treatment. ELISA assays were used to detect IL-18, IL-1β, TNF, IL-6 and IL-10 secretion in PBMCs.

Transparent reporting form
