# Supplementary material for: Different CFTR modulator combinations downregulate inflammation differently in cystic fibrosis
Source: eLife. 2020 Mar 2;9:e54556. doi: 10.7554/eLife.54556 (PMC7062465; doi:10.7554/eLife.54556)
Supplement: Supplementary file 1. — Data are expressed as median and range. BMI: Body Mass Index; ppFEV: percent predicted forced expiratory volume, ppFVC: forced vital capacity, CRP: C-reactive protein. WBC: white blood count. [file elife-54556-supp1.docx]

| Drug | ivacaftor/lumacaftor (IVA/LUM) | | | ivacaftor/tezacaftor (IVA/TEZ) | | |
| --- | --- | --- | --- | --- | --- | --- |
| Month | 0 | 1 | 3 | 0 | 1 | 3 |
| Genotype | Phe508del/ Phe508del | | | Phe508del/ Phe508del | | |
| Female (n) | 6 (13) | | | 4 (8) | | |
| Age, years | **30** (23-41) | | | **41** (28-50) | | |
| ppFEV_1_ | **29**  (12-44) | **37**  (12-44) | **33**  (17-42) | **26**  (19-35) | **26**  (19-38) | **29**  (19-40) |
| ppFVC | **43**  (31-65) | **48**  (31-65) | **44**  (30-66) | **43**  (36-80) | **47**  (35-78) | **48**  (29-74) |
| Weight, kg | **55**  (48-90) | **55**  (48-90) | **55**  (47-92) | **58**  (48-73) | **59**  (50-75) | **58**  (47-75) |
| BMI | **20**  (16-28) | **20**  (18-23) | **20**  (18-29) | **22**  (21-24) | **21**  (20-24) | **22**  (21-24) |
| CRP mg/ L | **5**  (5-31) | **15**  (5-44) | **8.5**  (5-149) | **23**  (5-77) | **23**  (5-45) | **29**  (5-51) |
| WBC | **9.3**  (6.7-11.6) | **10.7**  (6.8-17.1) | **9.1**  (5.1-13.8) | **9.6**  (4.8-15.3) | **11.5**  (6.1-17.6) | **10.6**  (5.2-16.3) |
| Neutrophil | **6.2**  (5.2-10.1) | **8.8**  (4.9-12.7) | **6.6**  (3.2-14.1) | **7.0**  (2.9-12.8) | **9.6**  (3.5-15.9) | **8.3**  (2.6-13.7) |

Supplementary file 1

Supplementary file 1. Demographic and clinical characteristics for CF patients on ivacaftor/lumacaftor (IVA/LUM) and ivacaftor/tezacaftor (IVA/TEZ). Data are expressed as median and range. BMI: Body Mass Index; ppFEV: percent predicted forced expiratory volume, ppFVC: forced vital capacity, CRP: C-reactive protein. WBC: white blood count.
