# Supplementary material for: Different CFTR modulator combinations downregulate inflammation differently in cystic fibrosis
Source: eLife. 2020 Mar 2;9:e54556. doi: 10.7554/eLife.54556 (PMC7062465; doi:10.7554/eLife.54556)
Supplement: Supplementary file 2. — ELISA assays were used to detect IL-18, IL-1β, TNF, IL-6 and IL-10 secretion in PBMCs. [file elife-54556-supp2.docx]

Supplementary file 2

| Drug | ivacaftor/lumacaftor (IVA/LUM) | | | ivacaftor/tezacaftor (IVA/TEZ) | | |
| --- | --- | --- | --- | --- | --- | --- |
| Month | 0 | 1 | 3 | 0 | 1 | 3 |
| IL-18  (pg/ mL) | 35.9 | 41.1 | 40.3 | 70.4 | 56.5 | 40.0 |
| IL-1β  (pg/ mL) | 46.4 | 35 | 29.4 | 23.8 | 4.6 | 5.2 |
| TNF  (pg/ mL) | 12.1 | 16.0 | 16.3 | 13.7 | 29.4 | 13.7 |
| IL-6  (pg/ mL) | 8.2 | 8.1 | 11.2 | 7.8 | 3.7 | 4.4 |
| IL-10  (pg/mL) | 3.1 | 5.1 | 15.0 | 2.6 | 0.8 | 0.6 |

Supplementary file 2. Cytokine secretion in unstimulated CF PBMCs following IVA/LUM (n=13) or IVA/TEZ (n=8) treatment. ELISA assays were used to detect IL-18, IL-1β, TNF, IL-6 and IL-10 secretion in PBMCs.
