# Supplementary material for: Different CFTR modulator combinations downregulate inflammation differently in cystic fibrosis
Source: eLife. 2020 Mar 2;9:e54556. doi: 10.7554/eLife.54556 (PMC7062465; doi:10.7554/eLife.54556)
Supplement: Peer review history

# Decision letter

**DOI:** 10.7554/eLife.54556.sa1

**Affiliations:** Radboud University Medical CentreNetherlands; Radboud UniversityNetherlands

In the interests of transparency, eLife publishes the most substantive revision requests and the accompanying author responses.

**Decision letter after peer review:**

Thank you for submitting your article "Different CFTR modulator combinations downregulate inflammation differently in cystic fibrosis" for consideration by *eLife*. Your article has been reviewed by Satyajit Rath as the Senior Editor, a Reviewing Editor, and two reviewers. The following individuals involved in review of your submission have agreed to reveal their identity: Rebecca Coll (Reviewer #1) and Siroon Bekkering (Reviewer #2).

The reviewers have discussed the reviews with one another and the Reviewing Editor has drafted this decision to help you prepare a revised submission.

Summary:

The manuscript provides additional evidence that systemic and cellular inflammation is of importance in CF. The study highlights the cellular anti-inflammatory effects of potent pharmacological inhibitors. The reviewers consider the data convincing and the subject generally compelling, but feel that the manuscript is difficult to follow and needs significant editing and re-organisation. Also, the mechanistic aspects should be discussed in more detail.

Essential revisions:

1) The grammar and sentence construction need to be improved throughout the manuscript.

2) The layout of the figures is not helpful to the reader. There is a rationale for grouping the two treatments in Figure 2 and Figure 3, but this makes comparing the data quite difficult as the reader to look between the two figures. We recommend that the data are grouped in a way that follows the description in the text.

3) Figure 2A and B appear to have been partially mislabelled – 2A should be IL-18 and 2B should be IL-1beta.

4) Although the authors measure caspase-1 activity as an additional non-cytokine based NLRP3 activation assay, in their previous publication the authors measured ASC specks in CF patient samples – did they do this in this study? This would be interesting data to include.

5) In subsection “NLRP3 inflammasome activation in patients receiving IVA/LUM or IVA/TEZ therapy” there is a description of mRNA levels in Figure 2, Figure 3, Figure 2—figure supplement 4, and Figure 3—figure supplement 3. There is a reference to NLRP3 mRNA which is shown in Figure 2K and Figure 3K but in the supplements NLRC4 mRNA appears to have been measured. This is however not discussed at all in the text. Have the genes been mixed up here?

6) There needs to be more discussion on the differential effects on IL-1beta between lumacaftor and tezacaftor. Is this related to the mechanism of action of these drugs?

7) How does the variability in cellular and systemic effects observed in the PBMCs of these patients correlate with the effects of these drugs in vivo? In the Introduction, the authors mention that not all CF patients benefit the same from these drugs and that efficacy varies. From Figure 2—figure supplement 1 it seems that these drugs did not have any effect on CF in these 3 months. Is that as expected? How long does it take for these drugs to see differences?

8) How do the authors think that the different drugs works intracellularly? The authors have tested one hypothesis but any mentioning of this hypothesis or other potential mechanisms is missing in the Discussion section. Are there any future directions to find out how it works? And does this have implications for drug efficacy in reducing CF?

9) The effects on serum IL-1beta are quite impressive. The lesson to be derived from this is that even when the intervention shows an effect on IL-1beta, this level of the cytokine is still in an inflammatory range. Hence a case could be made (in contrast with what the authors say in the Discussion section) that the clinical effect could be enhanced by combining treatment with anakinra. This point should be discussed in detail.

10) If samples and budget allow, it would be really interesting to perform RNA sequencing on the monocytes of these patients before and after treatment, also to find out pathway differences between the treatments.

# Reply: Author response

**DOI:** 10.7554/eLife.54556.sa2

Essential revisions:

1) The grammar and sentence construction need to be improved throughout the manuscript.

We have made significant changes to improve both grammar and sentence structure throughout the document.

2) The layout of the figures is not helpful to the reader. There is a rationale for grouping the two treatments in Figure 2 and Figure 3, but this makes comparing the data quite difficult as the reader to look between the two figures. We recommend that the data are grouped in a way that follows the description in the text.

We fully agree with the reviewers and have adjusted the graphs to reflect the description in the text. The main figures are now displayed under the following headings:

Figure 1. Cytokine secretion in NLRP3-stimulated monocytes, with a differential response between CF (homozygous Phe508del) and HC (healthy controls) following in vitro exposure to either IVA/LUM or IVA/TEZ.

Figure 2. Serum cytokine levels in patients with CF (homozygous Phe508del) following treatment with IVA/LUM or IVA/TEZ.

Figure 3. Cytokine secretion in NLRP3-stimulated CF immune cells isolated from patients with CF (homozygous Phe508del), following treatment with IVA/LUM or IVA/TEZ.

Figure 4. NLRP3-inflammasome activation in patients with CF (homozygous Phe508del) receiving IVA/LUM or IVA/TEZ treatment.

Figure 5. Sustainability of oral therapy (IVA/LUM or IVA/TEZ) on NLRP3-stimulated cytokine production in PBMCs isolated from patients on therapy for three months.

As a consequence of these changes, we have also altered the layout of some of the supplementary figures. They are displayed under the headings below. Supplementary file 1 and Supplementary file 2 remain unchanged.

Figure 2—figure supplement 1. Clinical measurements of patients with CF (homozygous Phe508del) following IVA/LUM treatment.

Figure 2—figure supplement 2 Clinical measurements of patients with CF (homozygous Phe508del) following IVA/TEZ treatment

Figure 2—figure supplement 3. Baseline serum cytokine levels in HC and CF (homozygous Phe508del) and corresponding baseline cytokine levels in NLRP3-stimulated HC and CF immune cells.

Figure 2—figure supplement 4 Individual therapy serum cytokine response in patients with CF (Phe508del/Phe508del) following IVA/LUM or IVA/TEZ treatment.

Figure 3—figure supplement 1. Individual therapy PBMC cytokine response in NLRP3-stimulated CF immune cells isolated from patients with CF (homozygous Phe508del) following treatment with IVA/LUM or IVA/TEZ.

Figure 4—figure supplement 1. mRNA expression in NLRP3-stimulated PBMCs isolated from patients with CF (homozygous Phe508del) following IVA/LUM or IVA/TEZ treatment.

3) Figure 2A and B appear to have been partially mislabelled – 2A should be IL-18 and 2B should be IL-1beta.

We have amended this in Figure 2.

4) Although the authors measure caspase-1 activity as an additional non-cytokine based NLRP3 activation assay, in their previous publication the authors measured ASC specks in CF patient samples – did they do this in this study? This would be interesting data to include.

Unfortunately, we did not measure ASC specks in this study. We have checked to see what samples we have remaining but we don’t have enough to produce a consistent data set. In our previous study (Scambler et al., 2019), ASC specks formation in CF patient samples correlated with both IL-1βand IL-18 cytokine secretion and intracellular caspase-1 activity. Due to the limited amount of sample available, we chose to look at caspase-1 activity in this study as a read out of NLRP3-inflammasome activation. The reviewer comments will help reshape the design of future studies which we are hoping to undertake as new drugs become available.

5) In subsection “NLRP3 inflammasome activation in patients receiving IVA/LUM or IVA/TEZ therapy” there is a description of mRNA levels in Figure 2, Figure 3, Figure 2—figure supplement 4, and Figure 3—figure supplement 3. There is a reference to NLRP3 mRNA which is shown in Figure 2K and Figure 3K but in the supplements NLRC4 mRNA appears to have been measured. This is however not discussed at all in the text. Have the genes been mixed up here?

We have amended the text to read NLRC4 rather than NLRP3. This was a typographical error.

6) There needs to be more discussion on the differential effects on IL-1beta between lumacaftor and tezacaftor. Is this related to the mechanism of action of these drugs?

We have expanded our discussion on CFTR modulators and their off-target effects. We have also added new references to support each statement.

“Both LUM and TEZ exert their clinical effects by increasing the processing and trafficking of mature CFTR protein to the cell surface. When combined with the potentiator, IVA, they partially rectify CFTR function and alter CFTR-ENaC coupling, which results in the inhibition of the elevated amiloride-sensitive sodium transport that is characteristic of CF (4, 5). Studies of nasal potential differences and rectal intestinal current measurements (ICM) studies suggest that dual combination therapy rescue Phe508del CFTR, by approximately 10% to 18% of normal, with both drugs having a similar effect on ion transport (24, 25). The disparity in IL-1β secretion was, therefore, surprising and may result from off-target effects, which have been reported with CFTR modulators. For instance, IVA can influence various solute carriers in vitro and reduce the stability of LUM-rescued Phe508del-CFTR (26). Clinically, IVA/LUM has also been associated with unexplained increased respiratory adverse events, which can occur relatively acutely after starting treatment. Symptoms include dyspnoea and chest tightness, features which have not been reported with IVA/TEZ (27). It might also be the case that pharmacokinetics of LUM and TEZ may differ.”

7) How does the variability in cellular and systemic effects observed in the PBMCs of these patients correlate with the effects of these drugs in vivo? In the Introduction, the authors mention that not all CF patients benefit the same from these drugs and that efficacy varies. From Figure 2—figure supplement 1 it seems that these drugs did not have any effect on CF in these 3 months. Is that as expected? How long does it take for these drugs to see differences?

We have offered an explanation as to why the systemic effects observed in PBMCs in our study don’t correlate with the patient’s clinical outcomes, in the text below, which has been added into the discussion. We have also added references to support each statement.

“During this study, combination therapy was only available through a Vertex compassionate use program, with treatment inclusion criteria including lung transplant assessment and a ppFEV1<40%, for at least two months. This is in contrast to reported phase 3 clinical trials which recruited stable patients with significantly higher lung function. In those studies, treatment over a 24-week period resulted in modest but significant improvements in lung function of around 3%, with reduced pulmonary exacerbations and a decrease in annual rate of ppFEV1 decline vs matched controls (28). Furthermore, in adults with severe obstructive lung disease receiving IVA/LUM, significant improvements in FEV1 can be absent in the first six months of treatment (29). While our study was not designed to investigate clinical efficacy, the absence of significant changes in clinical parameters, during our three month study period, was consistent with stable disease rather than a decline in health, as might be expected in a population with severe lung disease.”8) How do the authors think that the different drugs works intracellularly? The authors have tested one hypothesis but any mentioning of this hypothesis or other potential mechanisms is missing in the Discussion section. Are there any future directions to find out how it works? And does this have implications for drug efficacy in reducing CF?

In the Introduction we have modified the sentence which now reads:

“We observed CF-specific increases in IL-18, IL-1β, caspase-1 activity, in addition to ASC-speck release, that were all reversed by pre-treatment with epithelial sodium channel (ENaC) and NLRP3-inflammasome inhibitors (2, 3). In this paper, we examine the therapeutic potential of controlling inflammation with CFTR modulators. These small molecules are known to correct CFTR dysfunction and partially restore CFTR-mediated ENaC inhibitory activity (4, 5).”

Furthermore, in the Discussion section we have added the following statement as indicated in the response to comment 6:

“Both LUM and TEZ exert their clinical effects by increasing the processing and trafficking of mature CFTR protein to the cell surface. When combined with the potentiator, IVA, they partially rectify CFTR function and alter CFTR-ENaC coupling, which results in the inhibition of the elevated amiloride-sensitive sodium transport that is characteristic of CF (4, 5). Studies of nasal potential differences and rectal intestinal current measurements (ICM) studies suggest that dual combination therapy rescue Phe508del CFTR, by approximately 10% to 18% of normal, with both drugs having a similar effect on ion transport (24, 25).”9) The effects on serum IL-1beta are quite impressive. The lesson to be derived from this is that even when the intervention shows an effect on IL-1beta, this level of the cytokine is still in an inflammatory range. Hence a case could be made (in contrast with what the authors say in the Discussion section) that the clinical effect could be enhanced by combining treatment with anakinra. This point should be discussed in detail.

We fully agree with the reviewers. We have added the following sentence in the Discussion section.

“However baseline IL-1β levels remained elevated and additional targeting of the IL-1 receptor, could significantly enhance clinical effect, especially in patients on IVA/LUM where IL-1β was not significantly downregulated.”10) If samples and budget allow, it would be really interesting to perform RNA sequencing on the monocytes of these patients before and after treatment, also to find out pathway differences between the treatments.

We are unable, at this point, to collect further samples from patients before and after treatment of IVA/LUM and IVA/TEZ. However, RNA sequencing data will be invaluable to our future studies once we are able to extend our budget.
